# Dimercaprol Reprograms Intestinal Redox Homeostasis and Organelle Crosstalk to Combat Iron-Induced Gut Dysbiosis Through NRF2/HO-1 Signaling

**DOI:** 10.3390/antiox15030356

**Published:** 2026-03-11

**Authors:** Asad Khan, Zongliang Xiong, Iftikhar Ali Khan, Xiangyu Cheng, Qihui Luo, Lanlan Jia, Wentao Liu, Chao Huang, Zhengli Chen

**Affiliations:** 1 Laboratory of Experimental Animal Disease Model, College of Veterinary Medicine, Sichuan Agricultural University, Chengdu 611130, China; asad.khan@gu.edu.pk (A.K.); loveinchn@outlook.com (Z.X.); xiangyu16139@163.com (X.C.); iqhbiology@163.com (Q.L.); jialanlan@sicau.edu.cn (L.J.); liuwt1986@126.com (W.L.); 2 Key Laboratory of Animal Disease and Human Health of Sichuan Province, College of Veterinary Medicine, Sichuan Agricultural University, Chengdu 611130, China; 3 Department of Food Nutrition & Health, School of Food & Pharmaceutical Engineering, Zhaoqing University, Zhaoqing 526061, China; ifti_vet@szu.edu.cn

**Keywords:** iron-induced gut dysbiosis, mitochondrial dysfunction, oxidative stress, dimercaprol, NRF2/HO-1 pathway

## Abstract

Gut disorders are largely caused by iron-induced microbial dysbiosis. Excess iron disrupts barrier integrity by inducing oxidative stress, leading to impaired cellular processes. The determination of therapeutic compounds that can reduce iron-induced damage and maintain gut cellular integrity is still a top objective. Dimercaprol (DP) represents a novel iron-chelating strategy for the treatment of iron-induced gut disorders. A chronic iron-overload model was established in mice via intragastric gavage of ferric citrate (FC) (286 mg/kg BW) for 16 weeks. Similarly, IPEC-J2 cells were exposed to FC (50 µmol/L) for 24 h. DP was used as a mechanistic probe to elucidate the pathways involved in iron-induced toxicity. Cells were transfected with or without NRF2 siRNA and exposed to DP post-FC. Colonic contents were assessed via metagenomics and metabolomics. Both in vivo and in vitro experiments were analyzed through a multifaceted analysis, Western blot, RT-qPCR, ELISA, transmission electron microscopy and immunofluorescence assays. Thiols in DP protect gut cells from damage by boosting their natural antioxidant defenses via the NRF2/HO-1 pathway. The DP mechanism of action is multifaceted, including enhancement of barrier integrity, protecting mitochondrial structure and function, suppression of inflammation and endoplasmic reticulum (ER) stress and restoration of gut microbial and metabolic homeostasis. These protective effects are mainly caused by the activation of the NRF2/HO-1 pathway, which makes DP a potential therapeutic agent for disorders caused by chronic gut injury induced by FC. DP provides strong protection against iron-induced gut damage by restoring organelle crosstalk, redox homeostasis and microbial–metabolic balance through NRF2/HO-1 signaling.

## 1. Introduction

Iron is an important micronutrient that promotes body growth and development. It supports several metabolic processes, oxygen transport and storage, protein metabolism, redox reactions, energy synthesis, electron transfer and antioxidant defense [1]. Both excess and insufficient iron can be harmful to health; thus, balanced levels are essential. In ferroptosis [2,3], a controlled form of iron-dependent lipid peroxidation-driven cell death [4], gastrointestinal dysfunction results from excess intestinal iron, which compromises the integrity of the epithelial barrier by causing oxidative stress and inflammation [5,6]. Changes in gut microbial and metabolic balance, ER stress and mitochondrial dysfunction [7] are all closely linked to ferroptosis [8,9,10]. Ferroptosis caused by iron-induced gut barrier disruption is a clinically significant factor. However, there are currently limited effective therapeutic options.

The gastrointestinal tract exhibits high metabolic activity, and sustained exposure to endogenous and dietary iron makes it extremely vulnerable to iron-induced oxidative injury [11]. The colon is an essential organ for the absorption of nutrients and the removal of wastes [9]. Maintaining the host microbiota requires a diversified gut flora, which is dependent on iron availability [12]. Excess metal ions are bound by heavy metal chelators to create complexes that the body can more readily eliminate [13,14]. Therefore, treatments that may simultaneously repair mitochondrial and ER function, reset the microbiome and restore gut integrity are more intriguing.

An essential defense mechanism, the keap1-NRF2 pathway, aids cells in identifying oxidative stress and initiates defensive reactions to stop damage [15,16]. It is commonly recognized that NRF2 controls the expression of important antioxidant enzymes, including HO-1, to preserve intracellular homeostasis. It is therefore a prospective target for the treatment of illnesses associated with elevated oxidative stress and inflammation [15]. Keap1 typically suppresses NRF2 activity by protecting cells from ROS. Thus, inhibiting Keap1 can increase NRF2 activation and is a prospective therapeutic strategy for creating novel antioxidant medications [17,18]. NRF2/HO-1 activity is frequently suppressed by ferroptosis, which increases vulnerability to oxidative stress, lipid peroxidation and damage to the mitochondria and ER. Thus, NRF2 signaling restoration has become a prospective therapeutic target for reducing inflammation and ferroptosis-related cell death [19].

DP demonstrates strong therapeutic potential for traumatic optic neuropathy, primarily by alleviating oxidative stress [20]. The Food and Drug Administration (FDA) approved drug DP [21], which has recently gained attention for its antioxidative and cytoprotective potential. However, its role in ferroptosis-related intestinal injury remains unknown. In this study, we evaluated the therapeutic effects and underlying mechanisms of DP using both in vivo and in vitro models of iron-overload-induced gut damage.

## 2. Materials and Methods

### 2.1. Animal Care, Maintenance and Experimental Design

Nine-month-old male C57BL/6 mice (30 ± 2 g) were purchased from Beijing Vital River Laboratory Animal Technology Co., Ltd. (Beijing, China). The experimental animal model is schematically illustrated in Section 3.1. Mice (n = 48) were divided into two groups. One group (n = 16) received a daily phosphate-buffered saline (PBS) via intragastric gavage with vehicle, while the other (n = 32) was given FC (286 mg/kg BW, product: HY-N1428C, MCE) daily for 16 weeks to induce chronic iron overload in mice [6,22,23]. GI functional tests were conducted during this period. Body weight and feed intake were routinely measured. The mice displayed significant weight loss and constipation. Next, the FC mice were allocated into two groups: a positive control FC group (n = 16) and a DP (n = 16)-administered treatment group (Section 3.1). Mice received daily intraperitoneal injections of DP (10 mg/kg body weight) (HY-B1285, MCE) for four weeks [21]. Intestinal morphology and overall gut function were assessed. Samples for experimental analysis were obtained by euthanizing the mice after four weeks of DP treatment.

### 2.2. Behavioral Tests

All tests were carried out using standardized procedures.

#### Sucrose Preference Test

After a 24 h fasting period, the mice were then given access to two bottles, PBS and a 2% sucrose solution. The volumes of both solutions were recorded after 6 h. Total fluid intake was expressed as the combined consumption of sucrose solution and PBS.

### 2.3. Gastrointestinal Function Test

#### 2.3.1. Total Gastrointestinal Transit Time

Eosin dye was dissolved in a 0.5% sodium carboxymethyl cellulose solution to obtain a final concentration of 6%. Each mouse received 0.3 mL of eosin solution by oral gavage, and gastrointestinal transit was assessed by measuring the time to the first appearance of eosin-stained feces.

#### 2.3.2. Colonic Motility

A 3 mm glass bead was gently inserted approximately 2 cm into the mouse colon using a glass rod. Colonic motility was evaluated by recording the time required for bead expulsion. The time from bead insertion to expulsion was recorded. Mice not expelling the bead within 20 min were excluded [24].

### 2.4. 16S Amplicon Library Preparation and Sequencing

Colonic contents were processed via an OMEGA Soil DNA Kit (M5635-2, OMEGA Biotek, Norcross, GA, USA) per the guidelines. The forward primer 338 F (5′-ACTCCTACGGGAGGCGCAGCA-3′) and the reverse primer 806 R (5′-GGACTACHVGGGTWTCTAAT-3′) sequences were used to amplify and target the bacterial 16S rRNA gene V3-V5 region. The microbiome bioinformatic data were analyzed through QIIME2 2019.4, with some minor modifications [25]. Following the demux plugin demultiplexing of the raw sequence data, primers were cut via the cutadapt plugin [26]. The DADA2 plugin system was used to filter the quality, denoise, merge, and remove chimeras from the sequence [27]. Alpha diversity indices at the ASV level were computed via QIIME2. The structural variation in the microbial community among each sample was displayed by principal coordinate analysis (PCoA) and detected via beta diversity. The linear discriminant analysis effect size with default parameters was used to analyze the taxa differentially abundant among each group [28]. Under accession numbers, the sequence read archive (SRA) sequencing raw data are shown in Supplementary Table S1.

### 2.5. Liquid Chromatography–Mass Spectrometry (LC-MS)

Colonic contents were collected from mice and immediately frozen in liquid nitrogen. In order to obtain supernatant, 50 mg samples were homogenized in methanol and centrifuged at 12,000 rpm for 15 min at 4 °C. Thermo Scientific ultra-high-resolution LC-MS, using an Orbitrap Exploris 120 with a Vanquish Flex Column for separation(Thermo Fisher Scientific, Waltham, MA, USA), was used to profile untargeted metabolites. Data were acquired in both positive and negative modes. Compound Discoverer^TM^ 3.3 combined software was used to identify and quantify metabolites.

### 2.6. Colon Histopathological Examination

Colon samples from mice were obtained and preserved in paraformaldehyde. The specimens were dehydrated, cleared and embedded in paraffin. They were then sectioned at 5 µm and deparaffinized. Colon section pathology was evaluated by H&E (Servicebio, Wuhan, China) and alcian blue/periodic acid–Schiff (pH 2.5) (G1562) staining. Iron-deposited stains in the colon were detected with a Prussian Perls blue staining kit and 3,3′-diaminobenzidine (Boster, Wuhan, China) [6].

### 2.7. Immunohistochemistry (IHC)

Histologically, IHC staining was performed to identify specific protein markers in the colon. The primary antibody was used to identify TNF-α (Table 1). A final evaluation of all the results was performed with the Media Cybernetics software programs Image-Pro Plus 6.0 and OLYMPUS (Version 2.4.2).

### 2.8. Immunofluorescence (IF) Assay

Colon tissue sections were dried at 65 °C for 2 h, then deparaffinized and rehydrated. The sections were subjected to antigen retrieval by boiling in citrate buffer for 10 min. Slides were incubated in blocking solution containing 10% PBS, 0.01 g/mL donkey serum, 0.1% BSA and 0.1% X-100 Triton. Sections were incubated overnight at 4 °C with primary antibodies (Table 1) diluted in PBS and 1% donkey serum, followed by incubation with secondary antibodies for 90 min. Samples were covered with a drop of DAPI (P36962, Invitrogen, Carlsbad, CA, USA) to detect nuclei, and then a coverslip was applied. Slides were imaged using an Olympus BS61VS microscope (Tokyo, Japan). Following treatment, IPEC-J2 cells were fixed with 4% paraformaldehyde, permeabilized with 0.5% Triton X-100 for 5 min and blocked with 5% BSA. Cells were incubated overnight at 4 °C with the primary antibodies listed in Table 1. Species-appropriate secondary antibodies were applied, and the subsequent procedures were carried out as described above.

### 2.9. Transmission Electron Microscopy (TEM)

Colon tissue was fixed in 0.1 M phosphate buffer (pH 7.4) with 2.5% glutaraldehyde and then post-fixed for one hour in 1% osmium tetroxide. The samples were cleaned with propylene oxide, dehydrated in a graded series (30–100%) of ethanol and embedded in epoxy resin. An ultramicrotome (Leica EM UC7) (Wetzlar, Hesse, Germany) was used to cut ultrathin sections (70 nm), which were then put on copper grids and stained with lead citrate and uranyl acetate. Sections were then examined using a transmission electron microscope (TEM, JEOL JEM-1400) (Akishima, Tokyo, Japan) operated at 80 kV, and images were captured with a digital camera.

### 2.10. Cell Culture, FC Exposure and DP Treatment

The intestinal porcine epithelial (IPEC-J2) cell line was generously provided by Dr. Feng Bin (Sichuan Agricultural University). DMEM/F12 media (Thermo Fischer Scientific) supplemented with 10% fetal bovine serum (FBS, Gibco, Carlsbad, Carlsbad, CA, USA) was used to cultivate the cells. The medium was supplemented with 1% penicillin–streptomycin (10,000 U/mL penicillin and 10,000 µg/mL streptomycin, Gibco) and incubated at 37 °C with 5% CO_2_. Cells were cultured for 24 h or until they reached 70% confluency, then treated with 50 μM FC for 24 h. Following PBS washing, cells were treated with DP at 0, 25, 50, 100 and 200 µM concentrations for 6 h, according to experimental requirements. The cell counting kit-8 (CCK-8, C0042, Beyotime, Shanghai, China) was used to assess cell viability in accordance with the manufacturer’s instructions. A microplate reader (Thermo Scientific varioskan^TM^ LUX) was used to detect absorbance at 450 nm, and the results were expressed relative to the control (CK).

### 2.11. RNA Interference and IPEC-J2 Transfection

NRF2-specific double-stranded siRNA was custom-synthesized by RiboBio Co., Ltd. (Guangdong, China). The primers were designed as follows: forward 5′-GCCCAUUGAUCUCUCUCAUTT-3′ and reverse 5′-AUCACACACAUGGGCTT-3′. IPEC-J2 cells were seeded into six-well plates. Lipofectamine 3000 (Invitrogen, Carlbad, CA, USA) was used to transfect cells with 100 nM NRF2 siRNA at 70% confluence. Transfections were performed in Opti-MEM medium according to the manufacturer’s manual.

### 2.12. Determination of mRNA Levels via Reverse Transcription–Quantitative PCR

The (200) (Foregene) animal tissue RNA isolation kit was used to extract total RNA from both cultivated IPEC-J2 cells and mouse colon tissues. The HiScript II Q RT SuperMix kit (Vazyme, Nanjing, China) was used to reverse transcribe RNA into cDNA in accordance with the manufacturer’s instructions. A NanoDrop 2000 spectrophotometer (Thermo Fischer Scientific) was used to measure the concentration and purity of RNA. Gene expression was quantified using quantitative real-time PCR. Reactions were conducted with a 2x Sybr-Green Real-Time PCR Easy Mix (Foregene) on an Applied Biosystems 7500 Fast Real-Time PCR system (Foregene, Chengdu, China) in accordance with the manufacturer’s instructions. Data were collected and analyzed using the Bio-Rad^®^ PCR system (Hercules, CA, USA). The relative gene expression data were standardized using β-actin. The 2^−ΔΔCt^ technique was used to calculate relative gene expression levels. Table 2 lists the primer sequences used for each target gene.

### 2.13. Quantification of Protein by Western Blotting

Western blot analysis was performed according to a previously published protocol [29]. Total proteins were isolated from colon tissues and IPEC-J2 cells by lysing the samples with radioimmunoprecipitation assay (RIPA) buffer. A bicinchoninic acid assay kit (Boster, Wuhan, China) was used to assess protein quantities in the presence of protease inhibitors (Merck, Darmstadt, Germany). Proteins were first resolved using SDS-PAGE and then transferred to PVDF membranes for immunoblotting. Membranes were blocked with skim milk for 1 h and then incubated overnight at 4 °C with the primary antibodies listed in Table 1. The proteins were incubated with enzyme-conjugated secondary antibodies for 1 h, and the resulting bands were visualized using a Millipore enhanced chemiluminescence (ECL) detection kit (Darmstadt, Germany).

### 2.14. Lactate Dehydrogenase (LDH) Test

The cytotoxic effects of the treatments were assessed by measuring LDH release from IPEC-J2 cells using the LDH cytotoxicity assay kit (C0016, Beyotime, Shanghai, China). A total of 5 × 10^3^ cells were plated in 96-well plates and incubated overnight, followed by treatments as mentioned in Section 2.10. We added 10 μL of LDH release reagent to the cells, collected the culture supernatant and measured LDH activity.

### 2.15. Measurement of Cytochrome C Release

After each treatment, the cell supernatant was collected, lysed with 50 μL of cold lysis buffer and centrifuged at 10,000× *g* for 5 min at 4 °C. Cytochrome c levels in the supernatants were assessed using a sandwich ELISA (Pig Cytochrome C, ELISA^®^ Kit, #E0594p; EIAab, Wuhan, China) in accordance with the experimental protocol and the manufacturer’s guidelines. The microplate reader mentioned in Section 2.10 was used to measure absorbance at 450 nm, and results were expressed as a percentage relative to the CK.

### 2.16. Oxidation and Antioxidant Indices: Quantitative Detection

The fluorogenic probe 2,7-dichlorofluorescein diacetate (DCFH-DA, S01105, Beyotime, Shanghai, China) was used to measure the amounts of intracellular ROS and malondialdehyde (MDA, S0131, Beyotime, Shanghai, China) in IPEC-J2 cells according to the manufacturer’s guidelines. The antioxidant enzyme (SOD-S0101, catalase-S0082, T-AOC-S0121, GSSG/GSH-S0053 and NADPH/NADP^+^-S0180) activities in the IPEC-J2 cells and colon tissues were quantified using (Beyotime, Shanghai, China) assay kits, in accordance with the manufacturer’s instructions. The microplate reader mentioned in Section 2.10 was used to assess the sample absorbance, and the corresponding data were computed using a standard curve.

### 2.17. Statistical Analysis

Data processing and analysis for both the 16S rRNA sequencing and untargeted metabolomics were performed on the Personalbio Genes Cloud. Other non-sequencing and sample data were analyzed using R package Ropls (v.1.4.2)) and GraphPad Prism (v.10.3.0), respectively. Normally distributed data were analyzed using paired t-tests and one-way analysis of variance (ANOVA), followed by Dunnett’s multiple comparisons tests for further pairwise analysis. Data are presented as ± standard deviation (SD). The significance of differences among groups is indicated as non-significant “ns”, “*” (*p* < 0.01), “**” (*p* < 0.001), “***” (*p* < 0.0001) and “****” (*p* < 0.00001). The data are representative of at least three independent experiments.

## 3. Results

### 3.1. DP Reactivates the Cytoprotective NRF2/HO-1 Pathway

**Figure 1:**
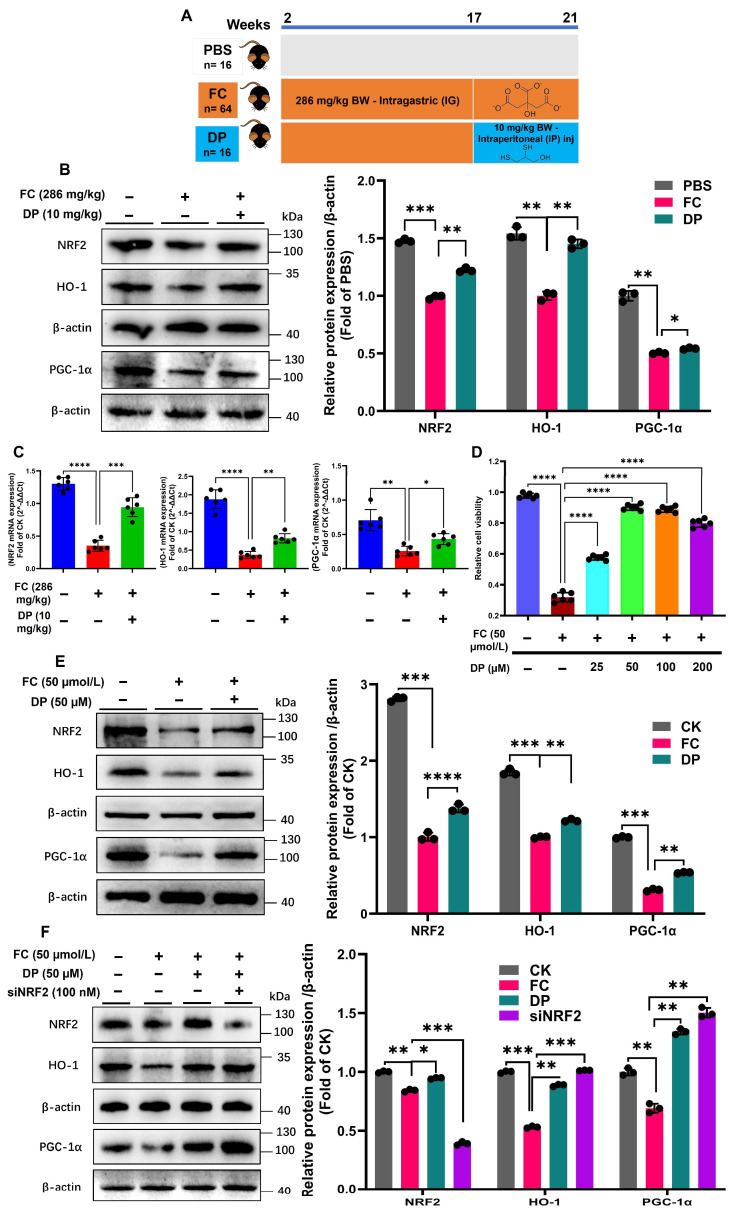

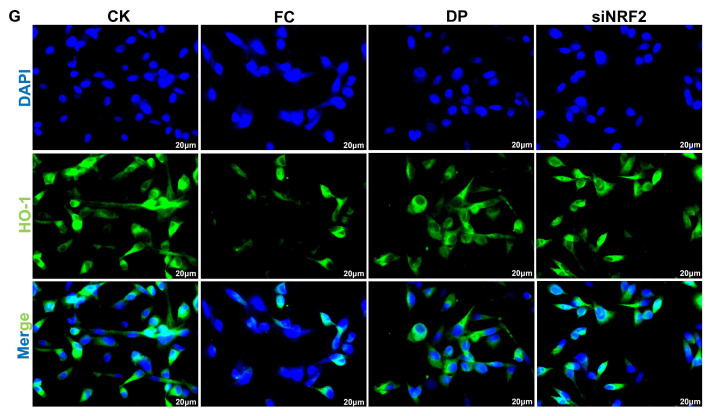
DP restores the NRF2/HO-1 antioxidant pathway impaired by iron overload. (**A**) Schematic representation of the in vivo chronic iron-overload model. (**B**) Western blot bands and corresponding quantification of NRF2, HO-1 and PGC-1α expression in colonic tissues from mice subjected to intragastric iron gavage (286 mg/kg body weight). (**C**) Relative mRNA expression of Nrf2/HO-1 in mouse colonic tissue. (**D**) Cell viability was assessed using the CCK-8 assay. DP enhanced the viability of FC (50 µmol/L) pre-stimulated IPEC-J2 cells in a dose-dependent manner, with maximal protection observed at 50–100 µM (viability ~0.89), representing nearly a threefold increase compared with the FC-only group (0.31). Data for histograms (**C**,**D**) were analyzed from six replicates (n = 6). (**E**) DP treatment increased NRF2/HO-1 and PGC-1α protein expression in IPEC-J2 cells, confirming pathway reactivation. (**F**,**G**) siNRF2 knockdown in IPEC-J2 cells confirmed the reduction in NRF2 protein levels. However, DP still increased HO-1 and PGC-1α expression, indicating a potential NRF2-independent mechanism of HO-1 and PGC-1α induction, demonstrated by Western blotting and immunofluorescence. Each experiment was repeated three times independently. The histograms (**B**,**E**,**F**) present the mean values ± SD of three replicates. Each group’s significant differences were compared with FC. * *p* < 0.01, ** *p* < 0.001, *** *p* < 0.0001, **** *p* < 0.00001.

To assess DP therapeutic potential, we induced chronic iron overload in mice by administering 286 mg/kg FC for 16 weeks (Figure 1A), a model previously established to trigger gut dysbiosis [9,22,23]. Under normal conditions, the NRF2/HO-1 pathway is a critical cytoprotective and antioxidant defense system [30]. Iron accumulation in mice hinders the function of the NRF2/HO-1 pathway, which is crucial for maintaining cellular homeostasis. Excess iron in the colons of mice impairs the NRF2/HO-1 pathway by depleting an essential protein [31]. However, DP administration restores the pathway function by normalizing the mitochondrial biogenesis protein levels in the colon (Figure 1B). Similarly, DP restores the mRNA levels disrupted by excess iron (Figure 1C). The viability of FC-pre-stimulated cells for 24 h [9] was measured after treatment with DP at 25, 50, 100, and 200 µM concentrations. Following FC exposure, DP increased cell viability in a dose-dependent manner. With a viability of (0.89), 50–100 µM DP treatment shows the maximum efficacy. This is almost three times higher than the FC-only control (0.31) (Figure 1D). DP decreased cell viability to (0.79) at a dosage of 200 µM, indicating toxicity [32]. These results suggest that activation of the cytoprotective NRF2/HO-1 signaling pathway is directly linked to the DP protective effect on cell viability. In IPEC-J2 cells, DP treatment raises NRF2 and HO-1 protein levels (Supplementary Figure S1A). According to these findings, the NRF2, HO-1 and PGC-1α protein levels are restored by DP (Figure 1E). Validation using siNRF2 in IPEC-J2 cells confirms the knockdown of NRF2 protein. Intriguingly, this reduction in NRF2 is accompanied by an increase in HO-1 and PGC-1α following DP treatment, suggesting a potential NRF2-independent mechanism for HO-1 induction (Figure 1F,G). These results are further supported by the mRNA expression levels of three key antioxidant regulators (NRF2, HO-1 and PGC-1α), which are significantly increased by DP treatment (Supplementary Figure S1B). These findings show that DP actively protects the cytoprotective NRF2/HO-1 signaling from iron-induced impairment in both in vivo and in vitro models.

### 3.2. Mechanistic NRF2/HO-1 DP-Dependent Pathway Enhances Antioxidants and Reduces Oxidative Stress

**Figure 2:**
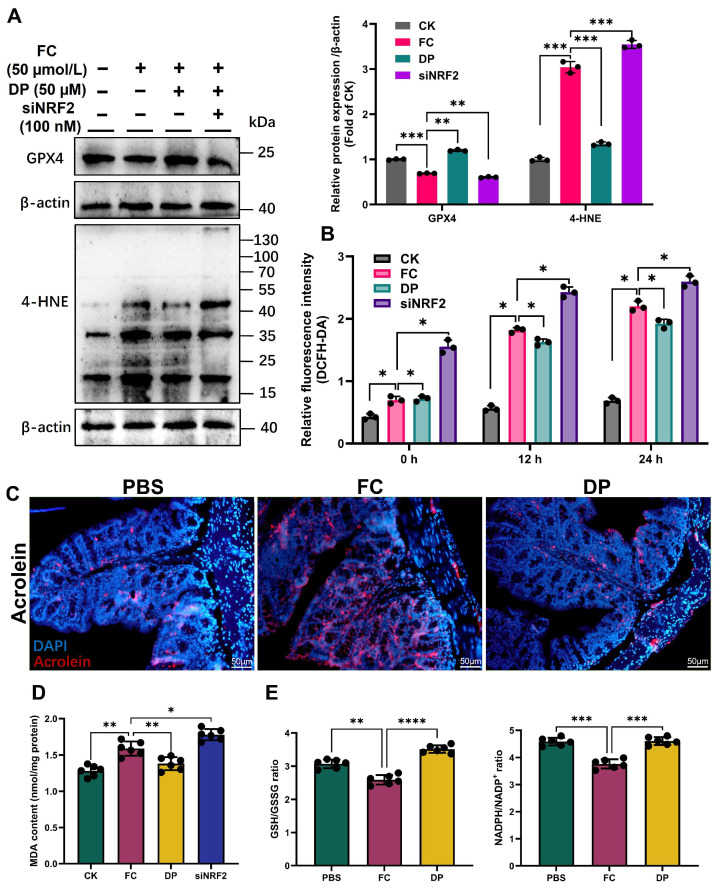

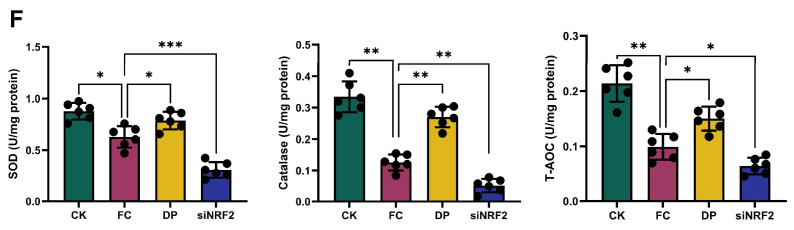
DP reduces oxidative stress by triggering the NRF2/HO-1 antioxidant regulatory mechanism. (**A**) Western blot analysis showing that DP reduces FC-induced 4-HNE and increases GPX4 protein expression in IPEC-J2 cells. These effects are abolished by NRF2 knockdown. (**B**) Time-course analysis of intracellular ROS (DCFH-DA) shows that DP markedly suppresses FC-induced ROS accumulation, whereas NRF2 knockdown abolishes this protective effect in IPEC-J2 cells. (**C**) Fluorescence intensity shows that DP scavenges acrolein accumulated in the colon following FC exposure. (**D**) Quantification of MDA content in IPEC-J2 cells confirms that DP-mediated reductions in lipid peroxidation depend on NRF2. (**E**) GSH/GSSG and NADPH/NADP^+^ ratios in the colon indicate enhanced cellular antioxidant defenses. (**F**) DP treatment elevates antioxidant capacity markers (SOD, catalase and T-AOC) in IPEC-J2 cells. Data were analyzed by linear regression to obtain the trendline, slope and intercept. The histograms (**D**–**F**) present the mean values ± SD of six replicates. Each group’s significant differences were compared with FC. * *p* < 0.01, ** *p* < 0.001, *** *p* < 0.0001, **** *p* < 0.00001.

To verify the role of the DP induction NRF2/HO-1 pathway in the oxidative stress response, we examined both in vivo and in vitro intracellular ROS levels at different time points. Similarly, the reduction of 4-HNE protein and MDA content by DP is dependent on NRF2, as knocking down NRF2 restores high 4-HNE and MDA levels (Figure 2A,D). MDA, a marker of lipid peroxidation, is a hallmark of ferroptosis, an emerging form of regulated cell death [4]. Furthermore, we analyzed the intensity of DCFH-DA in cells treated with DP and siNRF2 after FC exposure over time. DP suppressed FC-induced ROS levels, an effect that was reversed by NRF2 knockdown (Figure 2B). DP scavenges harmful acrolein in the colon accumulated with FC exposure (Figure 2C). DP increases GPX4 protein levels by activating NRF2, an effect abolished by NRF2 knockdown (Figure 2A). DP improved the redox balance in the colon by increasing the GSH/GSSG and NADPH/NADP^+^ ratios (Figure 2E). Consistently, the rise in SOD, Catalase and T-AOC following DP therapy indicates a boost in the cellular antioxidant response (Figure 2F). These findings show that DP enhances the cellular antioxidant response by activating the NRF2 pathway, which lowers oxidative stress and increases the expression of antioxidant enzymes.

### 3.3. DP Restores Mitochondrial–ER Crosstalk Integrity via the NRF2 Pathway

**Figure 3:**
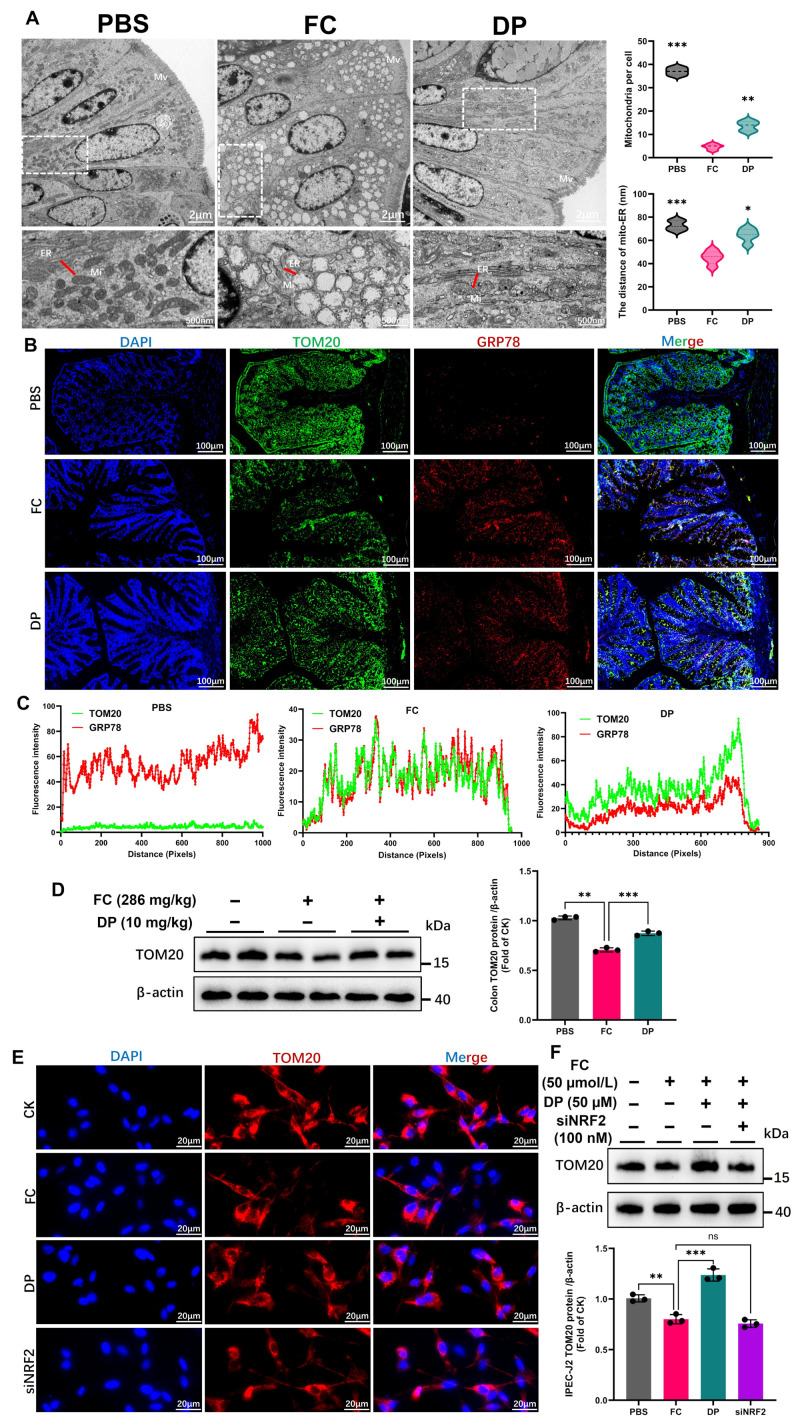
DP alleviates FC-induced ultrastructural damage and restores mitochondrial–ER integrity. (**A**) Transmission electron microscopy (TEM) images of colonic tissue show that FC induces mitochondrial swelling, vacuolization, ER dilation, and loss of microvilli, whereas DP treatment restores normal organelle morphology and mitochondrial number. Abbreviations in white represent the following: Mi = mitochondria, ER = endoplasmic reticulum, Mv = microvilli, and red solid lines indicate the distance between Mi and ER. Mi–ER distance was quantified by measuring the nearest contact sites between Mi and the ER. Data were analyzed using independent biological replicates (n = 6). (**B**) Immunofluorescence analysis demonstrates that DP treatment reduces GRP78 expression in the colon and its colocalization with the mitochondrial membrane TOM20. (**C**) Quantification of GRP78 and TOM20 contact sites shows that FC disrupts organelle spacing, while DP treatment re-establishes physiological proximity. Distances between colocalization contact sites were quantified in pixels using OlyVIA.Ink (Version 4.2). (**D**) Western blot band intensities and quantification show that DP rescues TOM20 protein in the colon. (**E**,**F**) Fluorescence intensity analysis and Western blotting demonstrate that DP restores TOM20 protein expression. This restorative effect is abolished following NRF2 knockdown, indicating that NRF2 is required for DP-mediated mitochondrial protection in IPEC-J2 cells. The histograms (**D**,**F**) present the mean values ± SD of three replicates. Each group’s significant differences were compared with FC. ns, non-significant, * *p* < 0.01, ** *p* < 0.001, *** *p* < 0.0001.

This study uses TEM to examine the effects of FC on the cellular architecture of the colon. The analysis shows swollen and vacuolated mitochondria and ER, indicating impaired function and disrupted crosstalk between these organelles (Figure 3A). The reduction in the number of mitochondria is restored by DP, which also reverses the loss caused by FC (Figure 3A). Based on fluorescence intensity and protein abundance (Figure 3B,D), FC induces a marked loss of the mitochondrial membrane protein TOM20, which is effectively restored by DP treatment. In contrast to FC, DP decreases the abnormal proximity between mitochondria and the ER (Figure 3A,C). GRP78, a key marker of ER stress, was overexpressed in response to FC, indicating ER dysfunction. DP not only reduced GRP78 levels but also promoted the physical separation of the ER from mitochondria, as reflected by its dissociation from TOM20 (Figure 3C), suggesting restoration of mitochondrial–ER crosstalk and alleviation of ER stress. It also restores TOM20 fluorescence intensity and protein levels in IPEC-J2 cells. However, NRF2 knockdown diminishes this restorative effect (Figure 3E,F). FC impairs gut cells by damaging their mitochondria and ER, primarily by activating the NRF2 pathway to restore cellular homeostasis.

### 3.4. DP Alleviates Iron-Induced Barrier Dysfunction, Mitochondrial Loss and Inflammation

**Figure 4:**
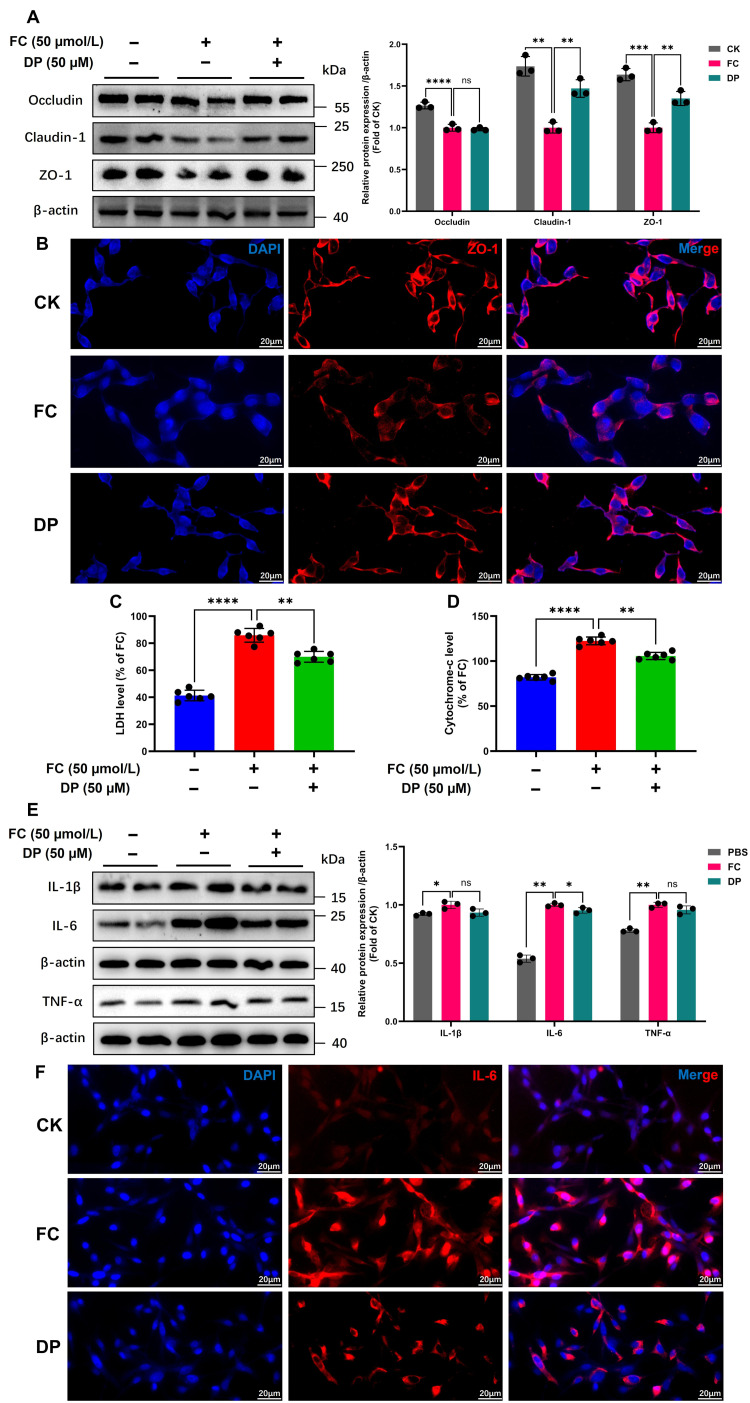
DP protects intestinal epithelial barrier integrity, preserves mitochondrial function, and suppresses inflammation under ferroptotic stress. (**A**) Western blot band intensities and quantification of occludin, claudin-1 and ZO-1 in IPEC-J2 cells. (**B**) Immunofluorescence assay represents ZO-1 localization in IPEC-J2 cells. (**C**,**D**) Cytosolic LDH and mitochondrial cytochrome c levels in IPEC-J2 cell supernatant 24 h after DP treatment. Data were analyzed by linear regression to obtain the trendline, slope and intercept. Histogram data were analyzed from six replicates (n = 6). (**E**) Western blot band intensities and quantification of IL-1β, IL-6, and TNF-α in IPEC-J cells. (**F**) Immunofluorescence staining of IL-6 in IPEC-J2 cells. Each experiment was repeated three times independently. The histograms (**A**,**E**) present the mean values ± SD of three replicates. Each group’s significant differences were compared with FC. ns, non-significant, * *p* < 0.01, ** *p* < 0.001, *** *p* < 0.0001, **** *p* < 0.00001.

Using an in vitro model of IPEC-J2 cells, we investigated the mechanisms of ferroptosis [33]. High-dose iron increases intestinal permeability by disrupting tight junctions via oxidative stress and inflammation [3,34]. The tight junction protein ZO-1, which localizes at cell–cell contacts in normal cells, was degraded and its junctional localization was disrupted by FC exposure, blurring cell boundaries. DP treatment reversed the damage by restoring ZO-1 protein expression and re-establishing well-defined cell borders (Figure 4B). In particular, DP improved the integrity of ZO-1, which had been compromised by FC in colon and IPEC-J2 cells (Supplementary Figure S2A) (Figure 4B). DP treatment significantly restored the expression of claudin-1 and ZO-1 in FC-treated IPEC-J2 cells, whereas occludin showed an increasing trend that did not reach statistical significance (Figure 4A). Consistent with these findings, DP similarly restored the mRNA expression of these three tight junction genes (Supplementary Figure S2C). The presence of epithelial tight junction proteins shows that the cytosolic barrier is intact and functioning. Subsequent analysis revealed that DP attenuated the significant rise in LDH release caused by FC (Figure 4C). The function of DP in controlling cytochrome c release was then examined. DP inhibits the release of mitochondrial cytochrome c (Figure 4D), indicating attenuation of mitochondrial outer membrane permeabilization and mitochondrial loss. Intestinal homeostasis disruption exacerbates inflammation brought on by long-term iron overload [6]. Moreover, DP significantly reduced FC-induced IL-6 protein levels, while IL-1β and TNF-α showed a decreasing trend that did not reach statistical significance (Figure 4E). Using an immunofluorescence assay, IL-6 was detected in colon tissue to evaluate the anti-inflammatory effect of DP (Figure 4F). In a mouse model of iron-induced colon, DP decreases inflammation by lowering TNF-α levels to corroborate in vitro results (Supplementary Figure S2B). Furthermore, DP treatment led to a significant decrease in the mRNA expression of the key pro-inflammatory cytokines IL-1β, IL-6, and TNF-α (Supplementary Figure S2D). Collectively, these results indicate that DP is a potent therapeutic agent that protects intestinal epithelial cells by reinforcing the physical barrier, preserving mitochondrial integrity, and attenuating the inflammatory response.

### 3.5. DP Recovers the NRF2/HO-1 Pathway by Mitigating the ERS

**Figure 5:**
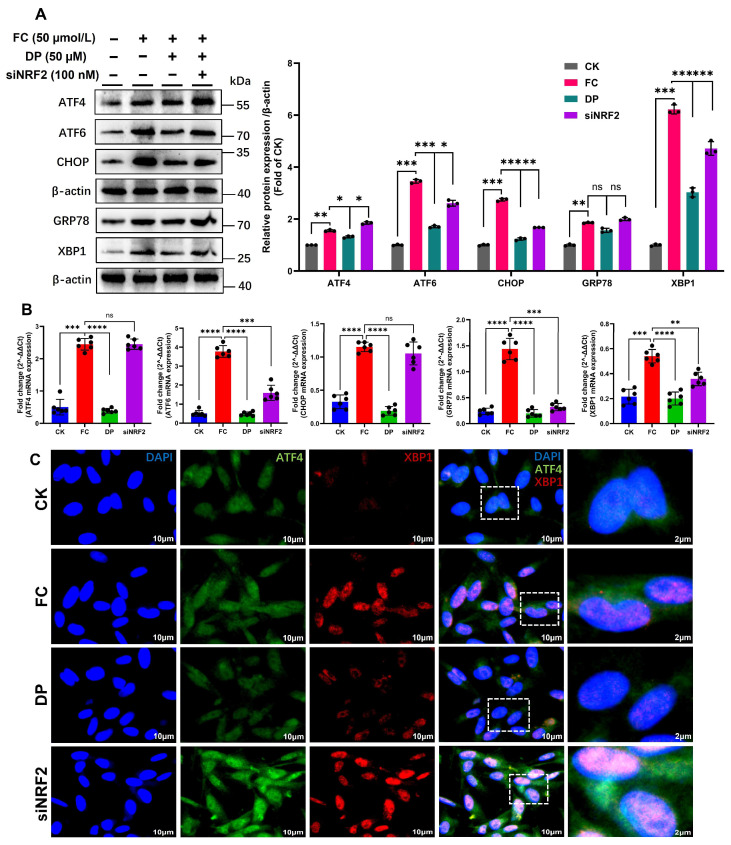
DP-dependent defense against FC-induced ER stress in IPEC-J2 cells is mediated by NRF2/HO-1 signaling. (**A**,**B**) Western blot band intensities and quantitative analyses indicate that FC exposure induces mitochondrial dysfunction and activates ER stress. This is evidenced by the notable increase in both protein and mRNA levels of canonical ER stress markers (ATF4, ATF6, CHOP, GRP78 and XBP1). DP attenuated this response, reducing the expression of these markers. In contrast, NRF2 knockdown abolished the protective effect of DP and further increased ER stress marker expression, indicating that DP-mediated ER stress suppression requires NRF2. (**C**) Fluorescence intensity confocal microscopy confirmed that NRF2 downregulation disrupts the colocalization of ER stress-related proteins, consistent with enhanced ER stress. Data are presented as mean ± SD for histogram (**A**) three replicates and (**B**) six replicates. Each group’s significant differences were compared with FC. ns, non-significant, * *p* < 0.01, ** *p* < 0.001, *** *p* < 0.0001, **** *p* < 0.00001.

Mechanistic role of the NRF2/HO-1 pathway linking mitochondrial and endoplasmic reticulum damage in FC-exposed IPEC-J2 cells. Mitochondrial damage induces endoplasmic reticulum stress (ERS). These findings demonstrate the significant upregulation of five canonical ERS markers, ATF4, ATF6, the pro-apoptotic transcription factor CHOP, the chaperone GRP78, and the spliced form of XBP1 (Figure 5A,B). After elucidating the mechanism by which DP regulates the NRF2/HO-1 pathway, we now examine whether NRF2 downregulation affects the expression of downstream effector molecules of key ERS genes in vitro. DP alleviates ERS by reducing the protein and mRNA levels of key markers, including ATF4, ATF6, CHOP, GRP78, and XBP1. Conversely, NRF2 knockdown increased their expression (Figure 5A,B). Similarly, confocal microscopy confirms that NRF2 downregulation disrupts the localization of ER stress-related proteins (Figure 5C). These findings collectively show that the NRF2/HO-1 pathway acts as an essential molecular bridge that reduces ER stress caused by FC. The ability of DP to suppress ER stress is mechanistically dependent on NRF2. Knockdown of NRF2 not only abolishes this protective effect but also markedly exacerbates the ER stress response.

### 3.6. DP Treatment Alleviates Ferroptosis by Reshaping the Gut Metabolome

**Figure 6:**
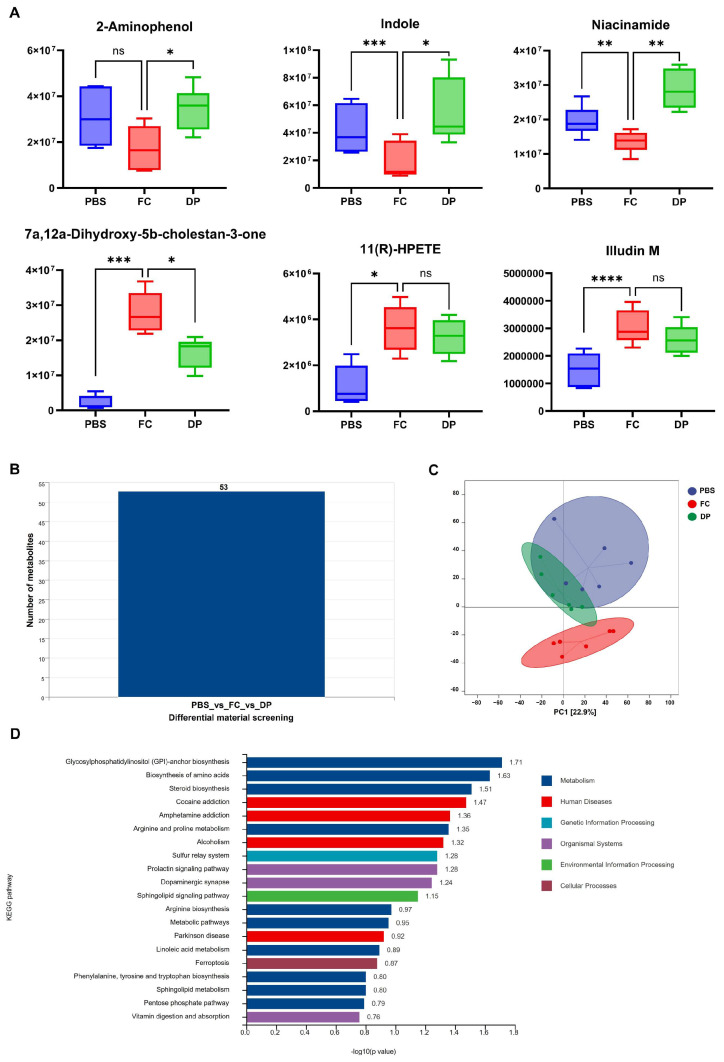
DP treatment reshapes the gut metabolite profile disrupted by iron overload. Untargeted LC-MS-based metabolomic profiling of colonic content. (**A**) Histogram of differential metabolite expression: 5-pentanoic acid, indole, niacinamide, 7a, 12a-Dihydroxy-5b-cholestan-3-one, 11(R)-HPETE, and Illudin M. Histogram data were analyzed from six replicates (n = 6). (**B**) Comparative analysis among PBS, FC, and DP groups identified 53 differentially expressed metabolites (DEMs). (**C**) Multivariate PCoA analysis among PBS, FC and DP groups clustered; the distance between DP and FC indicates restoration of the metabolic profile. (**D**) Pathway enrichment analysis of the DEMs across three groups revealed 20 significantly enriched pathways, primarily associated with metabolism, human disease, genetic information processing, organismal systems, environmental information and cellular processes. Each group’s significant differences were compared with FC. ns, non-significant, * *p* < 0.01, ** *p* < 0.001, *** *p* < 0.0001, **** *p* < 0.00001.

To understand the metabolite alterations in ferroptosis post-DP treatment, the primary and secondary metabolites in the colonic fecal samples were determined using the LC-MS platform. Based on the widely associated untargeted metabolomic analysis, we identified 405 metabolites. DP therapy significantly reverses the dysregulation of specific metabolites, including 5-pentanoic acid, indole and niacinamide (Figure 6A). High expression of 7a, 12a-Dihydroxy-5b-cholestan-3-one, 11(R)-HPETE and Illudin M is associated with ferroptosis (Figure 6A). Although DP broadly normalized the gut metabolite profile associated with iron overload, only one of the three was significantly reduced. A comparative analysis of PBS, FC, and DP groups revealed 53 differentially expressed metabolites (DEMs) (Figure 6B). Multivariate analysis reveals a clear separation of the FC group and a stronger association of the DP group with PBS (Figure 6C). Next, we screened the top 10 upregulated (Up) and top 10 downregulated (Down) differentially abundant metabolites with VIP ≥ 1, FC ≥ 1.5, or FC ≥ 0, and (*p* ≤ 0.05) values (Supplementary Table S2). Differential enrichment analysis across the six groups revealed 20 significant associations: 10 predominantly in metabolism, four in human disease, three in organismal systems, and one each in genetic, environmental, and cellular processes (Figure 6D). DP treatment reverses the metabolic changes caused by ferroptosis, returning the gut metabolite profile to normal (Figure 6C). The therapeutic effect of DP is demonstrated by its ability to normalize the dysregulated metabolite profile.

### 3.7. DP Repairs Gut Damage and Restores a Healthy Microbiome in Mice

**Figure 7:**
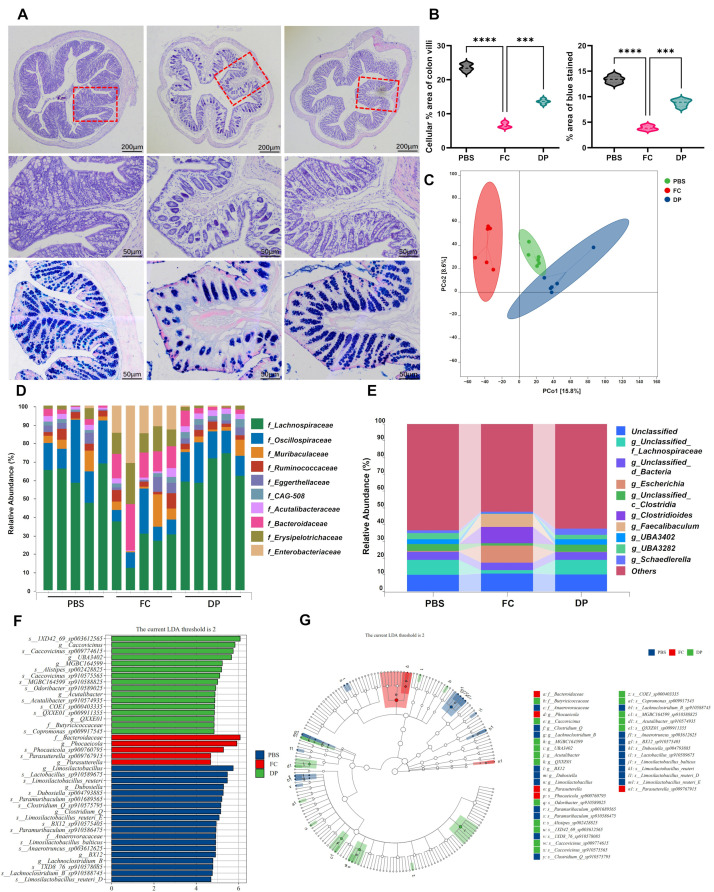
DP treatment alleviates iron-overload-induced gut injury, restores gut function and reverses dysbiosis. (**A**,**B**) Representative H&E and AB-PAS-stained colon sections with corresponding ImageJ (version 1.54) quantification, depicting the extent of histopathological alterations. (**C**) Beta diversity differences among groups were assessed using Principal Coordinates Analysis (PCoA) based on weighted UniFrac distance matrices. (**D**) Relative abundances of key bacterial families. (**E**) Differential genus-level abundances. (**F**) The histogram displays taxa with LDA scores greater than 2. Taxa with higher LDA scores represent those contributing more substantially to the differences observed among the groups. (**G**) LEfSe taxonomic cladogram. Colored nodes from the inner to the outer rings depict the hierarchical taxonomic structure from family to species. Non-significantly altered taxa are indicated in white. Circle diameter represents the relative abundance of each taxon. Each group’s significant differences were compared with FC. Data in panels (**C**) are shown as ±SD (n = 6) per group. *** *p* < 0.0001, **** *p* < 0.00001.

Histopathological changes in the colon were evaluated using H&E and AB-PAS staining. The colon tissue in the PBS group was characterized by abundant, densely packed, and orderly arranged cells. Conversely, the FC group displayed a marked deterioration, with a significant loss of cells, compromised tissue integrity, and a disordered architecture. The DP had significantly less tissue damage, better structural integrity and more mucous cells than the FC-induced mice (Figure 7A,B). The therapeutic potential of DP was evaluated using a series of behavioral and gut function tests. Similarly, gut functional assays revealed that DP significantly improved sucrose preference (Supplementary Figure S3A). However, there were no significant improvements in gut transit time, bead expulsion time, small intestine and colon length relative to the FC (Supplementary Figure S3B–E). According to 16S rRNA sequencing, FC is linked to gastrointestinal dysfunction by showing a significant disruption in gut community structure. This alteration was reversed in the DP, whose gut bacteria returned to a baseline state using weighted UniFrac analysis (Version 1.9) (Figure 7C). The *Enterobacteriaceae* (*p* < 0.003), *Erysipelotrichaceae* (*p* < 0.08) and *Bacteroidaceae* (*p* < 0.005) families were more abundant in the FC gut (*p* < 0.05), although DP treatment substantially lowered their abundances (*p* < 0.04) (Figure 7D). Furthermore, to validate these results at the genus level, we found that *Escherichia, Clostridioides* and Faecalibaculum abundances were high in the FC-induced mice (Figure 7E). In addition, a rich population of the genera Unclassified_f_Lachnospiraceae, Unclassified_c_Clostridia, *UBA3402* and *UBA3482* was observed in the PBS and DP (Figure 7E). LefSe analysis identified gut bacteria differentially abundant between the PBS and FC-induced groups. Using a linear discriminant analysis (LDA) threshold of >2.0, we identified several bacterial taxa as key biomarkers. PBS showed nineteen discriminative features, including g_limosilactobacillus, s_lactobacillus_sp910589675, s_limosilactobacillus_reuteri, g_dubosiella, s_dubosiella_sp004793885, s_paramuribaculum_sp001689565, s_clostridium_Q_sp910575795, g_clostridium_Q, and s_limosilactobacillus_reuteri_E, which were the most abundant. The bacterial family Bacteriodaceae, particularly the species Phocaeicola sp900760795 from the genus Phocaeicola, was the main among five key discriminative features in FC. The DP group was dominated by sixteen taxa, including s_1XD42_69_ssp003612565, g_Caccovicinus, s_Caccovicinus_sp009774615, g_UBA3402, g_MGBC_164599, s_Alistipes_sp002428825 and s_Caccovicimus_sp910575565 (Figure 7F,G). DP treatment demonstrates significant therapeutic efficacy in a mouse model of iron-overload-induced gut damage. It effectively restores colon tissue integrity and normalizes the gut microbiome by reversing the dysbiosis, particularly by suppressing pathogenic bacterial families and enriching beneficial taxa.

## 4. Discussion

Chronic iron overload induces ferroptosis-associated oxidative stress and damages the intestinal epithelial barrier, leading to gut microbiota dysbiosis [9,22,23]. The NRF2/HO-1 pathways play a dual role in ferroptosis. They typically protect against iron-dependent lipid peroxidation, but chronic or excessive activation can paradoxically promote ferroptosis. While NRF2-induced HO-1 reduces oxidative stress, sustained HO-1 activity increases labile Fe^2+^, thereby facilitating ferroptotic cell death. According to our findings, the NRF2/HO-1 cytoprotective pathway is impaired by long-term FC exposure. These results are in line with earlier findings showing that too much intracellular iron induces oxidative stress and destabilizes NRF2, which hinders antioxidant defense signaling [19,35,36,37,38,39]. These results align with prior reports demonstrating that pharmacological NRF2 activation improves epithelial barrier and reduces iron-driven oxidative stress [40,41,42,43]. The NRF2 knockdown shows that DP can also increase HO-1 expression via a largely NRF2-independent mechanism, which is consistent with other regulatory routes for HO-1 induction that have been documented previously [44]. Collectively, these results suggest that DP protects the NRF2/HO-1 signaling from iron-induced disruption in epithelial cells.

The aforementioned results indicate that DP protects against FC-induced oxidative stress by reactivating the cytoprotective NRF2/HO-1 pathway. The synchronized suppression of intracellular ROS and restoration of antioxidant defenses emphasize NRF2 as a key regulator of cellular redox homeostasis. These results align with previous studies demonstrating that NRF2 is an essential transcription factor that modulates cytoprotective genes in response to oxidative stress [39,45]. NRF2 knockdown markedly attenuates the antioxidant effects of DP, indicating that DP-mediated redox regulation is NRF2 dependent [19,35,46,47]. These findings show that DP therapy confers intestinal protection against FC-induced oxidative injury by activating the NRF2 pathway and reestablishing redox balance [48].

Excess iron accumulation significantly affects the mitochondrial and ER structure in gut cells, according to ultrastructural studies. These changes reflect a breakdown in normal mitochondria–ER coupling, leading to defective cellular communication and metabolic homeostasis [49]. Crucially, DP greatly corrects iron-induced structural defects in organelles, restoring mitochondria and microvilli. The therapy seems to restore appropriate communication between mitochondria and the ER by strengthening the integrity of the mitochondrial membrane. These findings indicate that mitochondria-associated membranes (MAMs), which are critical for the stability of epithelial cells, are mediated by maintaining physiological connections between mitochondria and the ER. To control lipid and calcium flow, mitochondrial dynamics, metabolic activity and stress adaptive pathways, MAMs coordinate mitochondria–ER interactions [49,50]. By normalizing MAM-associated signaling and restoring mitochondria–ER architecture, DP reduces gut damage by inhibiting the pathogenic cascade that causes tissue damage. Despite the limitation that mitochondrial membrane potential was not directly measured. This reestablishing of inter-organelle crosstalk helps heal the epithelial barrier, reduces ER stress and restores mitochondrial function.

These findings align with recent studies indicating that ferroptosis destabilizes gut epithelial architecture and exacerbates inflammation [51,52]. FC exposure diminishes intestinal barrier integrity by decreasing tight junction protein expression and facilitating the LDH and mitochondrial cytochrome c release. DP therapy successfully maintains the integrity of tight junctions, protects the potential of mitochondrial membranes and reduces epithelial damage. Ferroptosis is initiated by disruptions in iron metabolism, which increase lipid peroxidation and weaken antioxidant defense mechanisms [6,35,53]. DP therapy successfully reduces inflammation and rebuilds the permeability barrier.

Our findings reveal that FC exposure provokes concurrent ER stress and mitochondrial dysfunction in IPEC-J2 cells, as evidenced by the drastic elevation of important markers linked to ER stress. Collectively, these findings demonstrate that mitochondrial loss contributes to ER stress, promoting maladaptive mitochondria–ER crosstalk that exacerbates cellular stress [54]. Mechanistically, DP promotes cellular resilience by activating the NRF2/HO-1 pathway, which is a master regulator of antioxidant and electrophilic stress responses [45]. Activating this pathway reduces ER stress, indicating that NRF2 is an important regulator of ER homeostasis [55]. Importantly, loss of NRF2 abolished DP-mediated protection and significantly elevated ER stress, indicating NRF2 as a central regulator of cellular dysfunction induced by ER stress. These findings are consistent with previous research indicating that NRF2 stabilizes ER function via regulating chaperones, redox homeostasis and mitochondria–ER crosstalk, ultimately preventing CHOP-dependent apoptosis [56,57]. These findings strongly suggest targeting NRF2 signaling as a treatment strategy for gut disorders caused by mitochondria–ER stress responses.

These results support the growing hypothesis that effective treatment of complicated gastrointestinal disorders requires multimodal strategies that can repair the gut essential microbiota and metabolites [58,59]. Primary and secondary metabolites were significantly altered by FC-induced gut dysbiosis, with 7a, 12a-Dihydroxy-5b-cholestan-3-one, 11(R)-HPETE and illudin M being the most elevated. Key metabolites, such as 2-aminophenol, indole [60,61], and niacinamide [62], were normalized by DP, which suggests that the host microbial metabolic homeostasis has been restored. These findings are consistent with studies showing that indole and indole-derived metabolites participate in microbiome-mediated anti-ferroptotic pathways [61]. Niacinamide is a well-tolerated antioxidant that enhances cellular redox capacity and protects against oxidative stress-induced damage [62]. These findings align with reports demonstrating that gut-derived metabolites critically regulate both gut damage and epithelial integrity [63]. This metabolic recovery is in line with previous research showing that targeted modulation of the gut metabolites can effectively recover gut-associated disorders [64]. These results underscore DP’s potential as a treatment approach for ferroptosis-related disorders and indicate that it protects against iron-induced gut damage by restoring metabolic homeostasis.

Intestinal dysfunction is encouraged by iron overload, which drastically changes the composition of the gut microbiota and colonic metabolite profile [65]. It is essential to comprehend the causes and mechanisms of colonic inflammation during iron-induced damage. In line with these findings, iron-overload mice showed gut pathology and metabolic stress [66,67,68], including mucosal disruption, reduced motility and an abundance of inflammatory-associated taxa such as *Enterobacteriaceae* [69], *Erysipelotrichaceae* and *Bacteroidaceae* [70]. Defecation behavior and composition of the colonic microbial diversity changed noticeably after 16 weeks of FC gavage [6]. The FC-induced group showed a significant enrichment of pathogenic taxa, including *Escherichia*, *Clostridioides* and *Faecalibaculum*, which is consistent with their recognized harmful impacts on intestinal health [71,72,73]. Disruptions of the gut microbiota led to an increase in opportunistic pathogens and a decrease in beneficial bacteria, which interfere with normal microbial metabolic activities. Microbial activities that are vital for promoting and preserving host health are hampered by this imbalance [51]. In limitations, DP demonstrates potent protective effects in murine and IPEC-J2 models of iron-induced ferroptosis; these may not fully reflect the complexity of human intestinal physiology. Second, DP alters the gut microbiome and metabolome, but direct causality between specific microbes, metabolites and ferroptosis remains to be demonstrated. Third, the long-term safety, pharmacokinetics and dose response profile of DP remain uncharacterized. Taken together, these findings identify DP as a promising therapeutic candidate for FC-induced gut diseases. DP protects against iron-induced intestinal damage by resetting microbiome balance, maintaining mitochondrial–ER integrity and reducing ferroptosis-associated oxidative stress and inflammation through activation of NRF2/HO-1 signaling.

## 5. Conclusions

Mechanistically, DP suppresses ferroptosis by reactivating NRF2/HO-1 signaling, thereby boosting antioxidant defenses, preserving mitochondrial–ER crosstalk and reducing ER stress. DP confers protection through NRF2-mediated regulation of lipid peroxidation and mitochondrial function. DP therapy successfully protects against chronic iron-overload intestinal damage by restoring barrier integrity, stabilizing gut microbiota and reshaping metabolic disruptions. Overall, these results identify DP as a potential therapy for ferroptosis-related gut disorders and underscore the NRF2/HO-1 pathway as a key target in iron-induced intestinal dysfunction.

## Figures and Tables

**Table 1:** List of antibodies used in Western blot, immunofluorescence and immunohistochemical analyses.

Antibodies	Source	Identifier	Application
Anti-NRF2	ZEN (Chengdu, China)	R380773	WB
Anti-HO-1	Proteintech (Wuhan, China)	10701-1-AP	WB, IF
Anti-PGC-1α	ABclonal (Wuhan, China)	A20995	WB
Anti-GPX4	ABclonal (Wuhan, China)	A25009	WB
Anti-4-HNE	ABclonal (Wuhan, China)	A26085	WB
Acrolein	Abcam (Waltham, MA, USA)	ab48501	IF
Anti-TOM20	Proteintech (Wuhan, China)	11802-1-AP	WB, IF
Anti-Occludin	Beyotime (Shanghai, China)	AF7644	WB
Anti-Claudin-1	Beyotime (Shanghai, China)	AF6504	WB
Anti-ZO-1	Beyotime (Shanghai, China)	AF8394	WB, IF
Anti-IL-1β	ABclonal (Wuhan, China)	A16288	WB
Anti-IL-6	Huabio (Hangzhou, China)	EM1701-45	WB, IF
Anti-TNF-α	Cell Signaling Technology (Danvers, MA, USA)	11948T	WB, IHC
Anti-GRP78	Proteintech (Wuhan, China)	11587-1-AP	WB, IF
ATF4	Huabio (Hangzhou, China)	ET1612-37	WB, IF
ATF6	Huabio (Hangzhou, China)	EM1701-94	WB
CHOP	Proteintech (Wuhan, China)	15204-1-AP	WB
XBP1	BOSTER (Wuhan, China)	PB9463	WB
Anti-β-actin	ABclonal (Wuhan, China)	AC026	WB
Alexa Fluor^®^ 488 Goat anti-Rabbit	Thermo Fischer Scientific (Waltham, MA, USA)	A-11070	IF (Green)
Alexa Fluor^®^ 594 Goat anti-Rabbit	Thermo Fischer Scientific (Waltham, MA, USA)	A-11012	IF (Red)
Alexa Fluor^®^ 488 Goat anti-Mouse	Thermo Fischer Scientific (Waltham, MA, USA)	A-11029	IF (Green)
Alexa Fluor^®^ 594 Goat anti-Mouse	Thermo Fischer Scientific (Waltham, MA, USA)	A-11032	IF (Red)

**Table 2:** Quantitative real-time PCR primer sequences.

Gene		Primer (5′–3′)	Accession Number
*β-actin (Mus Musculus)*	F R	AGAGGGAAATCGTGCGTGAC CAATAGTGATGACCTGGCCGT	NM_007393.5
*NRF2*	F R	CAGCCATGACTGATTTAAGCAG CAGCTGCTTGTTTTCGGTATTA	NM_010902.5
*HO-1*	F R	AGGTACACATCCAAGCCGAGA CATCACCAGCTTAAAGCCTTCT	NM_010442.2
*PGC-1a*	F R	GGATATACTTTACGCAGGTCGA CGTCTGAGTTGGTATCTAGGTC	NM_001402987.1
*IL-1b*	F	CCCCAGGGCATGTTAAGGAG	NM_008361.4
	R	TCTTGGCCGAGGACTAAGGA	
*IL-6*	F	CTTCCATCCAGTTGCCTTCTTG	NM_031168.2
	R	AATTAAGCCTCCGACTTGTGAAG	
*TNF-a*	F	ACGGCATGGATCTCAAAGAC	NM_013693.3
	R	GTGGGTGAGGAGCACGTAG	
*β-actin (Sus Scrofa)*	F	GGACTTCGAGCAGGAGATGG	XM_003357928.4
	R	GCACCGTGTTGGCGTAGAGG	
*NRF2*	F R	GCACCGTGTTGGCGTAGAGG TCCATGTCCCTTGACAGCAA	XM_003133500
*HO-1*	F R	GGCTGAGAATGCCGAGTT ATGTAGCGGGTGTAGGCGTGGG	NM_001004027.1
*PGC-1a*	F R	GAGATTCCGTATCACCACC CTTTCAGACTCCCGCTTCC	AB106108
*Occludin*	F	ACGAGCAGCAAAGGGATTCTTC	NM_001163647.2
	R	TCACACCCAGGATAGCACTCATT	
*Claudin-1*	F R	TGCCTCAGTGGAAGATTTACTCC TGGTGTTCAGATTCAGCAAGGA	NM_013693.3
*ZO-1*	F	AGTTTGATAGTGGCGTTGACAC	XM_005659811.1
	R	GCTGAAGGACTCACAGGAACA	
*ATF4*	F	ATGCCCTGTCGGGTATAGATGA	NM_001123078.1
	R	ATCCAACGTGGCCAAAAGC	
*ATF6*	F	GGGAGTGAGCTGCAGGTGTATT	XM_021089516.1
	R	TCTGCGGATGGCTTCAAAGA	
*CHOP*	F	GGAAATGAGGAGGAGTCAAAAACC	NM_001144845.1
	R	CTCAGTCAGCCAAGCCAGAGA	
*GRP78*	F R	TGGGAAAGAAGGTTACTCATGCA CTGGCGTTGGGCATCATT	X92446.1
*XBP1*	F R	CAGACTGCCAGAGACCGAAAGA TCTTCCAAATCTACCACTTGTTGCT	NM_001142836.1

## Data Availability

The original contributions presented in this study are included in the article and Supplementary Materials. Further inquiries can be directed to the corresponding authors.

## Supplementary Materials

The following supporting information can be downloaded at: https://www.mdpi.com/article/10.3390/antiox15030356/s1, Figure S1: DP enhances NRF2/HO-1 signaling expression in IPEC-J2 cells. Figure S2: DP reduces inflammation and restores tight-junction gene expression in iron-overload gut cells. Figure S3: Effects of DP treatment on behavioral performance and physiological parameters in iron-overload mice. Table S1: Sequence reads archive (SRA) accession, bioproject, and biosample numbers through quality screening. Table S2: Differentially expressed metabolites (DEMs) among the top 10 upregulated (up) and downregulated (down) genes were identified on the basis of variables with a VIP score of ≥1, fold change (FC), log2FC, and *p* value.

## Abbreviations

BW	Body weight
IPEC-J2	Intestinal porcine epithelial cells-jejunum 2
NRF2	Nuclear factor erythroid 2-related factor 2
siRNA	Small interfering ribonucleic acid
qRT-PCR	Quantitative reverse transcription polymerase chain reaction
ELISA	Enzyme-linked immunosorbent assay
HO-1	Heme oxygenase-1
MCE	MedChem express
ASV	Amplicon sequence variant
TNF-α	Tumor necrosis factor-alpha
BSA	Bovine serum albumin
DAPI	4′,6-diamidino-2-phenylindole
TOM20	Translocase of the outer mitochondrial membrane 20
GRP78	Glucose-regulated protein 78
ATF4	Activating transcription factor 4
CHOP	C/EBP homologous protein
XBP1	X-box binding protein 1
SOD	Superoxide dismutase
T-AOC	Total-antioxidant capacity
GSSG/GSH	Glutathione disulfide/reduced glutathione
NADPH/NADP	Reduced nicotinamide adenine dinucleotide phosphate/Nicotinamide adenine dinucleotide
AB-PAS	Alcian blue/periodic acid–Schiff
LefSe	Linear discriminant analysis effect size
PGC1-α	Peroxisome proliferator-activated receptor gamma coactivator 1-alpha
