# Supplementary material for: Dimercaprol Reprograms Intestinal Redox Homeostasis and Organelle Crosstalk to Combat Iron-Induced Gut Dysbiosis Through NRF2/HO-1 Signaling
Source: Antioxidants (Basel). 2026 Mar 11;15(3):356. doi: 10.3390/antiox15030356 (PMC13024590; doi:10.3390/antiox15030356)
Supplement: Supplementary file 1 [file antioxidants-15-00356-s001.zip › antioxidants-4144668-supplementary.pdf]

Supplementary tables

Supplementary Table S1. Sequence reads archive (SRA) accession, bioproject, and biosample numbers through quality screening.

SRA accession	BioProject	BioSample
SRR31938149	PRJNA1208258	SAMN46171817
SRR31938150	PRJNA1208258	SAMN46171816
SRR31938151	PRJNA1208258	SAMN46171815
SRR31938152	PRJNA1208258	SAMN46171814
SRR31938153	PRJNA1208258	SAMN46171813

Supplementary Table S2. Differentially expressed metabolites (DEMs) among the top 10 upregulated (up) and downregulated (down) genes were identified on the basis of variables with a VIP score of ≥1, fold change (FC), log2FC, and P value.

Class	PBS_VS_FC				FC_VS_DP				
	**VIP**	**FC**	**Log2FC**	**P-Val**	**VIP**	**FC**	**Log2FC**	**P-Val**	**DEM**
**2-Aminophenol**	1.71	1.71	0.77	0.08	1.82	1.94	0.95	0.02	Up
**Indole**	2.16	2.20	1.14	0.01	2.16	2.91	1.54	0.004	Up
**Niacinamide**	1.95	1.67	0.74	0.11	1.88	1.10	3.46	0.02	Up
**Dihydrothymine**	1.59	1.46	0.54	0.19	1.04	1.54	0.62	0.19	Up
**Pyrrolidonecarboxylic acid**	1.83	2.60	1.37	0.11	0.19	2.67	1.41	0.75	Up
**cis-4-Hydroxy-L-proline**	1.81	2.42	1.27	0.06	1.74	4.01	2.004	0.03	Up
**L-Isoleucine**	1.95	3.33	1.73	0.05	1.77	3.21	1.68	0.02	Up
**cis-4-Hydroxy-D-proline**	1.14	2.35	1.23	0.21	1.65	3.86	1.95	0.03	Up
**Hydroxyindole**	1.55	1.45	0.54	0.04	1.58	1.41	0.50	0.05	Up
**Chavicol**	0.49	1.15	0.20	0.88	0.70	0.75	-0.40	0.41	Up
**7a,12a-Dihydroxy-5b-cholestan-3-one**	0.51	0.80	-0.30	0.49	1.42	0.39	-1.33	0.10	Down
**11(R)-HPETE**	0.06	0.42	-1.22	0.900	1.71	0.02	-5.28	0.04	Down
**Illudin M**	0.67	0.98	-0.01	0.801	1.98	0.30	-1.71	0.007	Down
**16(R)-HETE**	0.60	0.87	-0.19	0.480	1.68	0.56	-0.83	0.04	Down
**Docosahexaenoic acid**	1.73	0.37	-1.41	0.047	1.08	0.53	-0.90	0.22	Down
**3-Dehydroecdysone**	1.52	0.19	-2.35	0.204	1.85	0.07	-3.76	0.02	Down
**2,22-Dideoxy-3-dehydroecdysone**	1.12	0.26	-1.90	0.342	1.64	0.12	-3.03	0.04	Down
**Acetaminophen**	0.76	0.71	-0.49	0.36	1.70	0.21	-2.23	0.08	Down
**DHHA**	1.34	0.54	-0.87	0.075	1.55	0.50	-0.98	0.10	Down
**D-Alanyl-D-serine**	0.33	0.86	-0.20	0.639	2.08	0.43	-1.21	0.01	Down

Supplementary figures

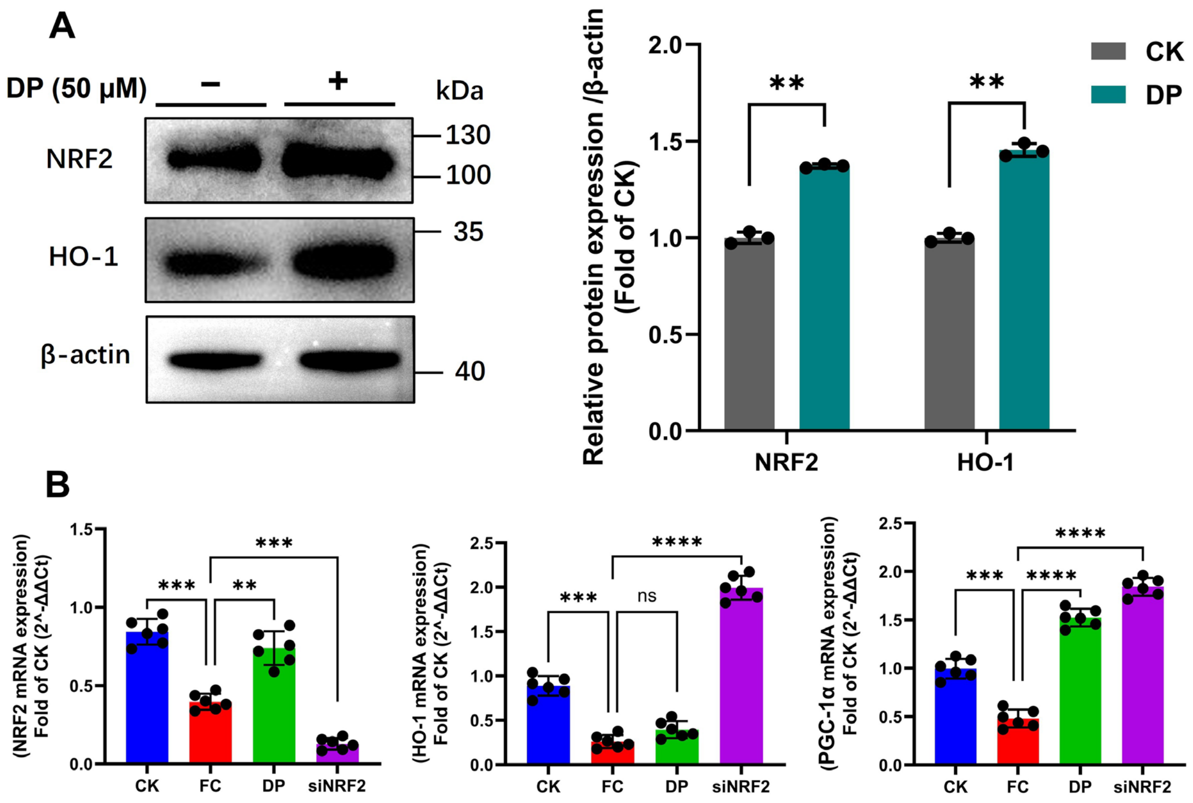

Supplementary Figure S1. DP enhances NRF2/HO-1 signaling expression in IPEC-J2 cells. **(A)** DP treatment increases NRF2 and HO-1 protein levels in FC-exposed IPEC-J2 cells. **(B)** RT-qPCR analysis demonstrated that DP treatment increased the mRNA expression of NRF2, HO-1, and PGC1-α, both in the presence and absence of NRF2. The histograms present the mean values ± SD of six replicates. Each group's significant differences were compared with FC.

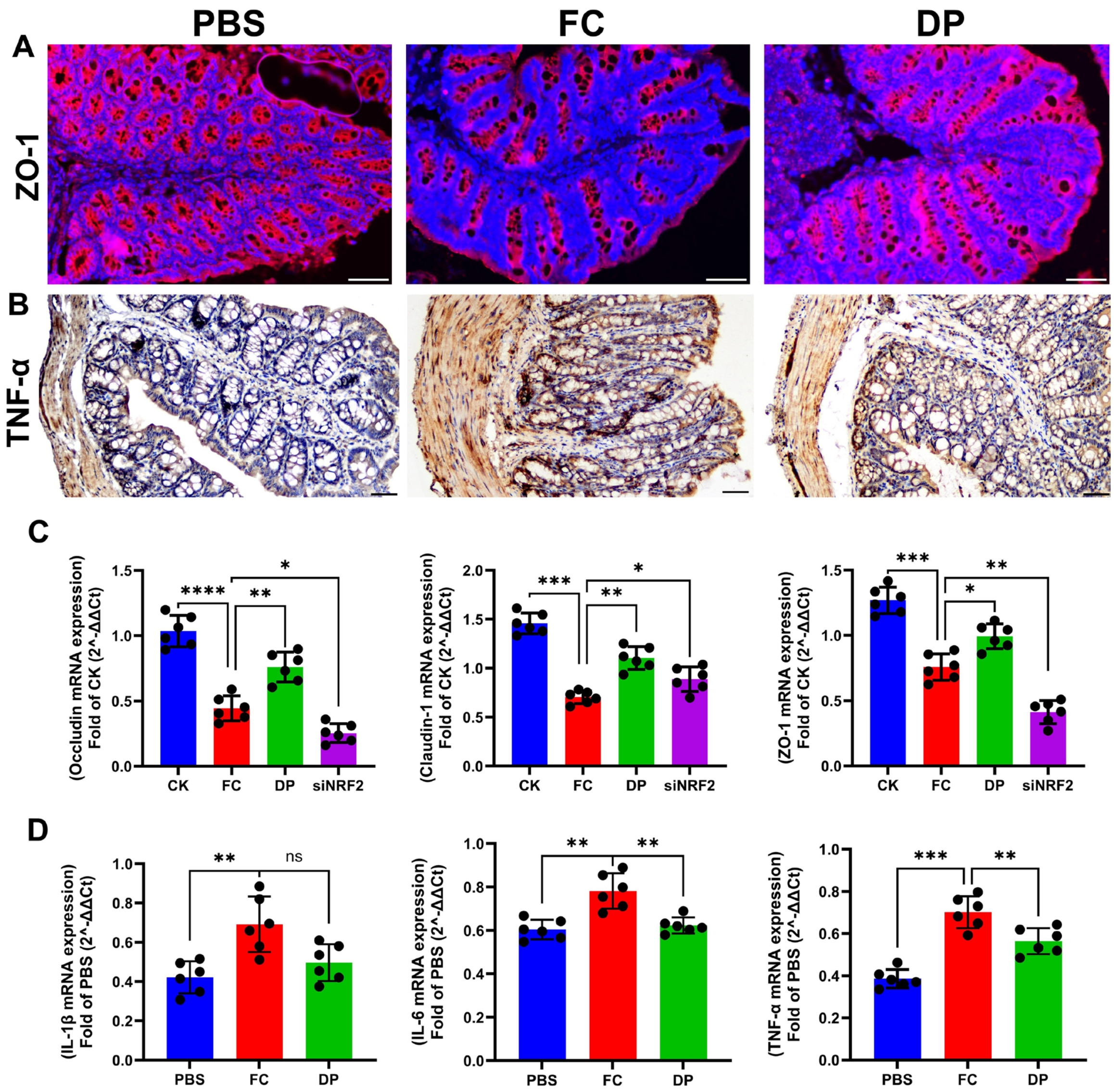

Supplementary Figure S2. DP reduces inflammation and restores tight-junction gene expression in iron-overload gut cells. **(A)** Immunofluorescence staining represents that DP restored the tight junction protein ZO-1 in colon. **(B)** Immunohistochemistry showing that DP treatment markedly decreases TNF-α levels in colonic tissue. **(C)** Relative mRNA levels of occludin, claudin-1, and ZO-1 show that DP reverses iron-induced transcriptional suppression in IPEC-J2 cells. **(D)** RT-qPCR analysis showing that DP significantly reduces IL-1β, IL-6, and TNF-α expression in iron-overloaded colon tissue. The histograms present the mean values ± SD of six replicates. Each group's significant differences were compared with FC.

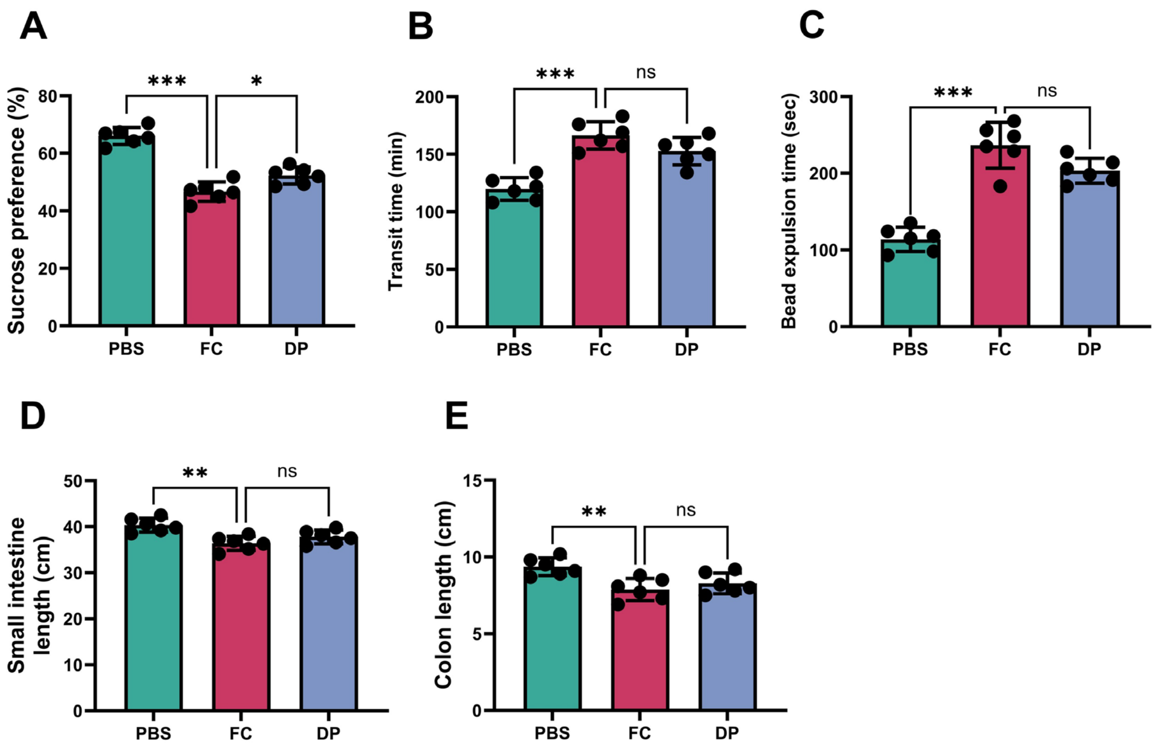

Supplementary Figure S3. Effects of DP treatment on behavioral performance and physiological parameters in iron-overload mice. **(A)** Assessment of sucrose preference test in PBS, FC and DP-treated mice. **(B)** Gastrointestinal transit time following DP treatment in iron-overloaded mice. **(C)** Bead expulsion time as an indicator of colonic motility in each group. **(D)** Small intestine and, **(E)** Colon length. The histograms present the mean values ± SD of six independent experiments. Each group's significant differences were compared with FC.
